# Designing and Analyzing the Structure of DT-STXB Fusion Protein as an Anti-tumor Agent: An *in Silico *Approach

**DOI:** 10.30699/ijp.2019.101200.2004

**Published:** 2019-09-22

**Authors:** Zeynab Mohseni Moghadam, Raheleh Halabian, Hamid Sedighian, Elham Behzadi, Jafar Amani, Abbas Ali Imani Fooladi

**Affiliations:** 1 *Applied Microbiology Research Center, Systems Biology and Poisonings Institute, Baqiyatallah University of Medical Sciences, Tehran, Iran *; 2 *Department of Microbiology, College of Basic Sciences, Shahr-e-Qods Branch, Islamic Azad University, Tehran, Iran*

**Keywords:** In silico modeling, Diphtheria toxin, Shiga-like toxin part B, Cancer therapy, Bioinformatics tools

## Abstract

**Background & Objective:**

A main contest in chemotherapy is to obtain regulator above the biodistribution of cytotoxic drugs. The utmost promising strategy comprises of drugs coupled with a tumor-targeting bearer that results in wide cytotoxic activity and particular delivery. The B-subunit of Shiga toxin (STxB) is nontoxic and possesses low immunogenicity that exactly binds to the globotriaosylceramide (Gb3/CD77). Gb3/CD77 extremely expresses on a number of human tumors such as pancreatic, colon, and breast cancer and acts as a functional receptor for Shiga toxin (STx). Then, this toxin can be applied to target Gb3-positive human tumors. In this study, we evaluated DT390-STXB chimeric protein as a new anti-tumor candidate via genetically fusing the DT390 fragment of DT538 (Native diphtheria toxin) to STxB.

**Methods:**

This study intended to investigate the DT390- STxB fusion protein structure *in silico. *Considering the *Escherichia coli *codon usage, the genomic construct was designed. The properties and the structure of the protein were determined by an *in silico* technique. The mRNA structure and the physicochemical characteristics, construction, and the stability of the designed chimeric protein were analyzed using computational and bioinformatics tools and servers. Hence, the GOR4 and I-TASSER online web servers were used to predict the secondary and tertiary structures of the designed protein.

**Results:**

The results demonstrated that codon adaptation index (CAI) of *dt390-stxB* chimeric gene raised from 0.6 in the wild type to 0.9 in the chimeric optimized gene. The mfold data revealed that the *dt390-stxB* mRNA was completely stable to be translated effectively in the novel host. The normal activity of the fusion protein determined by considering the secondary and tertiary structure of each construct. Energy calculation data indicated that the thermodynamic ensemble for mRNA structure was -427.40 kJ/mol. The stability index (SI) of DT390-STxB was 36.95, which is quite appropriate to preserve the stability of the construct. Ultimately, the DT390-STxB was classified as a steady fusion protein according to the Ramachandran plot.

**Conclusion:**

Our results showed that DT390-STXB was a stable chimeric protein and it can be recruited as a candidate of novel anti-tumor agents for the development of breast cancer treatment.

## Introduction

Tumors in cancer patients, which have been cured by surgery, chemotherapy, and radiotherapy return in numerous cases and they mostly metastasize (1, 2). The general therapy of cancer has been focused on the nonspecific practice of cytotoxic substances for many years to extinguish neoplastic cells. Yet, chemotherapy is confined via parallel impairment to natural tissues, which results in dose-limiting toxicities (3). The confined remedial index of cytotoxic drugs and the capacity of cancer cells to progress resistance to these manufactured medications is essential to expand novel instruments for aggressive malignancies alone or in combination with cytotoxic factors. Anti-tumor action of bacterial toxins has been recognized in numerous studies. Moreover, it has been assumed that the conjugated compounds of bacterial toxins and ligands kill several kinds of tumors (4-8). Thus, in this study we selected diphtheria toxin (DT) and the B-subunit of Shiga toxin (STxB) as anti-tumor agents. Native DT is composed of a 538-amino acid protein with three domains; part A is the enzymatically active domain, part B has a hydrophobic domain at the N-terminal portion, and the C-terminal portion of fragment B is the receptor-binding domain. DT intoxicates susceptible eukaryotic cells via receptor-mediated endocytosis (9). As soon as internalized within an acidic vesicle, the enzymatically active fragment A portion is released into the cytosol. Protein synthesis is repressed by part A-catalyzed adenine diphosphate (ADP) ribosylation of elongation factor 2, and eventually leads to cell death. This is an effective and capable procedure, likewise, the single molecule of DT can impede up to 2000 ribosomes/min in cell-free systems (10).

The Shiga toxins attached to the AB5 toxins, with an enzymatically active A segment and a nontoxic B segment, are responsible for binding to cellular receptors. The B moiety comprises of five similar B subunits (7.7 kDa) establishing a pentameric circle nearby the central pore in which the C-terminus of the A moiety is attached. Each B-subunit anchorages three determined binding sites that exactly interrelate with the glycosphingolipid (Gb3). So, each B moiety can possibly interact with up to 15 Gb3 molecules, leading to high-affinity binding (11). In humans, expression of Gb3 is limited to apparent cell types. Moreover, a distinct inclination towards Gb3 overexpression in tumor tissues compared with the normal tissue has been observed in several cancers such as pancreas, breast, and ovary and also in malignant meningioma, glioma, and acute non-lymphocytic leukemia (6, 11-17).

In this study, we designed and constructed a DT-STxB chimeric protein and its fusion protein linked by a hydrophobic linker specified as a candidate for cancer therapy. 

## Materials and Methods

**Designing the Chimeric Construct**

The amino acid sequences of DT and STxB proteins were retrieved from Uniprot database. The selected sequences for designing chimeric construct were DT (accession No. Q6KE85) and STxB (accession No. Q7BQ98). The DT consists of 193-amino acid N-terminal A-chain, the 342-amino acid B-chain with a hydrophobic translocation enhancing region, a disulfide bond among chains A and B with the deletion of 145-amino acid innate binding region. In order to construct DT390–STxB fusion, DT was fused to the amino acid sequence of STxB (21-89) as a binding fragment for the cellular receptors. Two parts of the fusion protein were linked using a hydrophobic GGGSGGGSGGGS amino acid linker. To optimize the synthetic gene, *in silico* analysis was performed using online databases such as Gene Bank codon database, the codon database, and Swiss-Prot reverse translation online tool; gene designer software was also recruited. Furthermore, several hydrophobic linkers were examined by the GOR4 tool (18) to separate two functional parts of the chimeric protein. The chimeric gene was designed for cloning and expression in *Escherichia coli* (*E.coli*) using the Java codon optimization tool (JCat) (http://www .jcat.de/ ) and optimizer web server (19, 20). VaxiJen server (www.ddg-pharmfac.net/vaxijen) (21) was recruited to predict the immunogenicity of the whole antigen. The chimeric gene was synthesized by Biomatik Company (Ontario, Canada).

**RNA Secondary Structure Analysis**

 The mRNA secondary structure of the chimeric gene was analyzed using the mfold server (http://mfold.rna. albany .edu/ ) (22).

**Chimeric protein confidants**

Physiochemical characterization, theoretical isoelectric point (pI), molecular weight, total number of positive and negative residues, extinction coefficient (E), instability index (II), aliphatic index (AI), and grand average hydropathy (GRAVY) of the chimeric protein were attained using Expasy ProtParam server (web.expasy.org/ protparam/) (23).

**Protein Secondary Structure**

Prediction of secondary structure of DT-STxB fusion protein sequence was made using GOR secondary structure prediction method version IV (http://gor.bb.iastate.edu/) (24). Furthermore, the functional characteristics of the designed protein such as secondary structure, the regions lacking normal structure, coiled-coil domains, sections with low-complexity, transmembrane (TM) helices, the solvent accessible surface area (SASA), and the sites with disulfide bridges were also assessed.

**3D Structure Prediction**

Further analysis of tertiary structural stability of the recombinant DT390-STxB protein was done by the I-TASSER online server (http://zhanglab.ccmb.med. umich.edu/I-TASSER) (25-27), Phyre version 0.2 (http://www.sbg.bio.ic.ac.uk/phyre2 ) (28), and online web servers separately, which generates 3D models along with their confidence score (C-Score). 

Energy minimization was confirmed by examining the stability of 3D structure of the chimeric protein recruiting Swiss-PdbViewer (aka DeepView) (29). To assess the availability of the amino acids in the protein, the online ASA program was used (30).

**Evaluation of Tertiary Structure**

Following generating 3D model, the Swiss PdbViewer that includes a version of the GROMOS96 43B1 force field was performed for energy minimization. The ProSA-web, Z-scores, and Procheck Ramachandran plot (mordred.bioc. cam.ac.uk/~rapper/rampage.php) were performed for structural evaluation and stereo chemical analyses (31). Also, the alignment of the query and template sequences and the assessment of the generated models were carried out recruiting the Swiss-PdbViewer. Furthermore, the solubility of the DT390-STxB fusion protein was evaluated by PROSO online software (https://prosa.services.came .sbg.ac.at/prosa.php ) (32).

**Prediction of Antigenic Properties of the Chimeric Protein**

VaxiJen web server (www.ddg-pharmfac.net/vaxijen) (21) was applied for predicating antigenic properties of the chimeric protein based on the physicochemical properties.

## Results

**Fig. 1:**
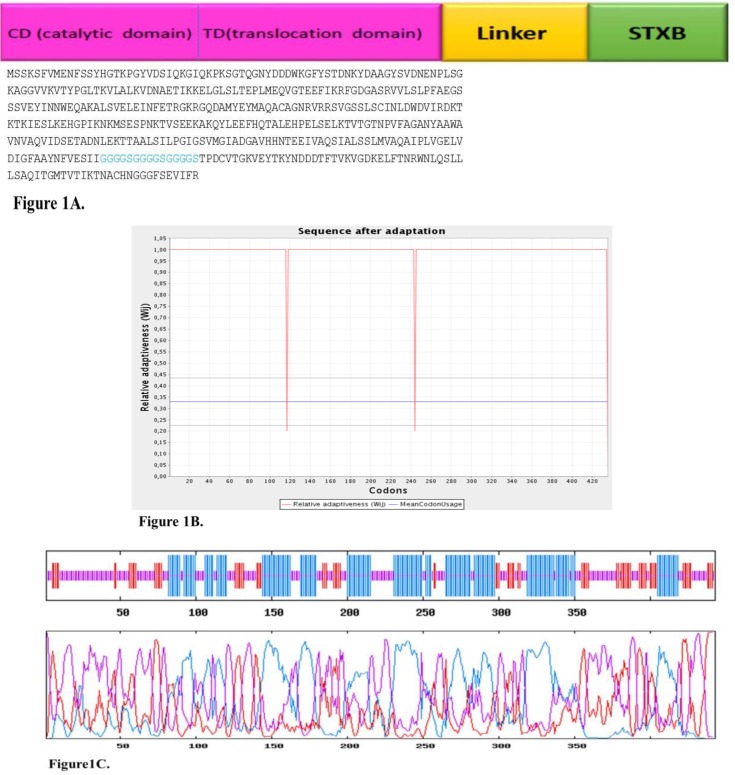
**A**. Sequence alignment and schematic model of hypothetical chimeric protein, which has exposed the construct of DT390 (catalytic and translocate domains) and STxB bound together by the GGGGSGGGGSGGGGS appropriate linker for expression in *E. coli*. **B. **Adaptation of codon usage intermittent repartition. The red line demonstrates the codon usage for every codon, which is present in the gene. The blue line shows the mean codon usage in *E. coli* which was assessed for any recognized gene of this organism. The grey lines above and under the blue line display the standard deviation for this mean codon usage in the *E. coli*. **C.** Graphical picture of secondary elements in chimeric DT390-STxB protein. The solubility supplies have been categorized by the main polarity and hydrophobic properties of residual patterns. These patterns have exposed that the mean residue accessible surface area (ASA) have given a high solvent convenience value

INNO The design and analysis of chimeric protein properties of 390 residues from N-terminal of DT (lacking 21 amino acids of signal sequence from the N-terminal of the protein) was selected from online gene banks and fused to the amino acid sequence of STxB. Several hydrophobic linkers were tested to choose the top linker which sustains functionality of the standard structure of the two parts of the recombinant protein. Finally, GGGGSGGGGSGGGGS sequence was designated as the linker to maintain the flexibility of the construct (Figure 1A).

Afterwards, the amino acid sequence was back-translated and nucleic acid codons were adjusted based on the codon, considering *E. coli* as the expression host. The chimeric gene exposed a codon adaptation index (CAI) of 0.62 in comparison to the wild type gene that was 50.15%. The GC% codon usage bias in *E. coli* was augmented by upgrading GC% and CAI to 50.15% (GC% of *E. coli* is nearby 50) and 0.62, respectively. The CAI of >0.8 was considered statistically significant in terms of high gene expression level (Figure 1B).

Furthermore, the essential restriction enzyme sites (*Eco*RI* and Hin*dIII) were introduced at the ends of the sequence for cloning purposes.

**The mRNA Structure Prediction**

The secondary structure of the *dt390-stxB* mRNA was predicted using the online software mfold. To specify the anticipated folding of the designed gene, a genetic algorithm-based RNA secondary structure prediction was associated with comparative sequence analysis. The folding of 5' terminal region of the gene had the same structure as the bacterial gene. The minimum free energy (MFE) was estimated for RNA secondary structure; all 29 structural elements recovered in this study had the same the folding as RNA constructs. The obtained data demonstrated that the mRNA had the sufficient stability for the effective translation in the novel host. 

**Fig. 2:**
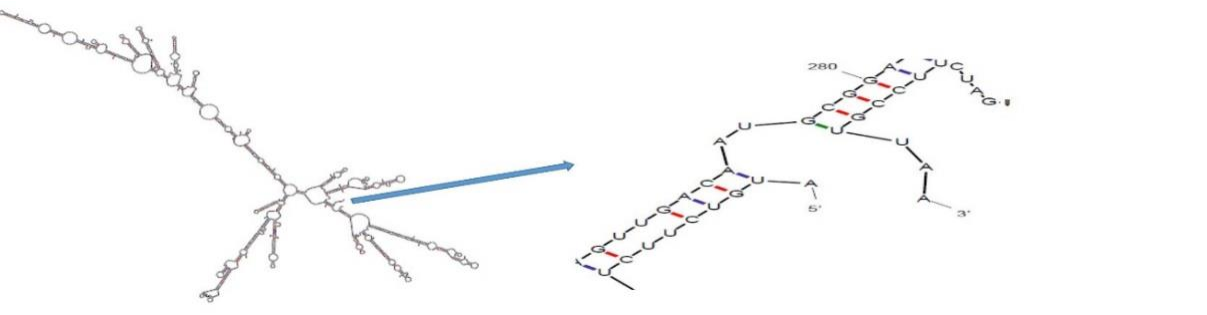
The mRNA secondary structure of *dt390-stxB* prediction. Predicted structure has a pseudoknot at 5′ site of mRNA. (ΔG=-427.40 kJ/mol) in mfold tool

 Results are demonstrated in Figure 2 and Table 1. The free energy of thermodynamic cooperative units associated with this structure was ΔG=-427.40 kJ/mol.

Random coils established to be recurrent 189, 81 extended strand and 189 alpha helix (Table 2 and Figure 1C).

**Chimeric Protein Properties**

ProtParam web server was used to reveal the physiochemical properties of the DT390-STxB sequence. The number of amino acids was found to be 443, the average molecular weight was 53 kDa, and the pI was 5.11. In addition, the total number of positively charged residues (Arg+Lys) was 42 and total number of negatively charged residues (Asp+Glu) was 56. The instability index of DT390-STxB was computed by the ProtParam web server, which appeared to be 36.95 (Table 3, 4).

**Table 1:** Thermodynamic details related to 5’ end of chimeric construct DT390-STxB mRNA. According to the minimum free energy (ΔG) of the 5' end of chimeric mRNA, their initial ATG is constrained in hairpin loop structure

Information(DT390-STXB)	ΔG(DT390-STXB)	Structural Element
**3 base pairs.**	-5.50	Helix
**Closing pair is U335-A340**	5.40	Hairpin loop
**External closing pair is U2-A274**	-2.10	Stack
**External closing pair is G3-C273**	-2.20	Stack
**External closing pair is U4-A272**	-2.40	Stack

**Table 2:** Arrangement of secondary structure of origin and chimeric protein

Protein	Extended Strand		Random Coil
**DT390-STXB**	81	173	189
**DT390**	52	160	147
**STxB**	26	14	29

Data are presented as %.

**Table 3:** Parameters calculated by ExPSy’s ProtParam tool

GRAVY	AI	II	EC	+R	-R	TpI	Mw	Sequence length
**-0.312**	78.13	36.95	49850	42	56	5.11	53645.38	443

*Mw, Molecular weight; T pI, Theoretical Isoelectric point; -R, Number of negative charged residues; +R, Number of positive charged residues; EC, Extinction coefficient at 280 nm; II, Instability index; AI, Aliphatic index; GRAVY, Grand Average Hydropathy.

**Table 4:** Amino acid composition of DT390-STxB chimeric protein

Amino acid	No. of residues	Percentage of residues	Amino acid	No. of residues	Percentage of residues
**Ala (A)**	34	7.7%	Phe (F)	15	3.4%
**Arg (R)**	10	2.3%	Pro (P)	13	2.9%
**Asn (N)**	24	5.4%	Ser (S)	39	8.8%
**Asp (D)**	22	5.0%	Thr (T)	30	6.8%
**Cys (C)**	4	0.9%	Trp (W)	5	1.1%
**Gln (Q)**	14	3.2%	Tyr (Y)	15	3.4%
**Glu (E)**	34	7.7%	Val (V)	35	7.9%
**Gly (G)**	47	10.6%	Leu (L)	31	7.0%
**His(H)**	7	1.6%	Lys (K)	32	7.2%
**Ile (I)**	23	5.2%	Met (M)	9	2.0%

However, by considering these details, the chimeric protein is classified as a stable protein. The estimated half-life of this recombinant protein was 30 h (mammalian reticulocytes, in vitro), >20 h (yeast, in vivo), and >10 h (*E. coli*, in vivo). The grand hydropathicity is considered to be -0.312.

**Figure 3:**
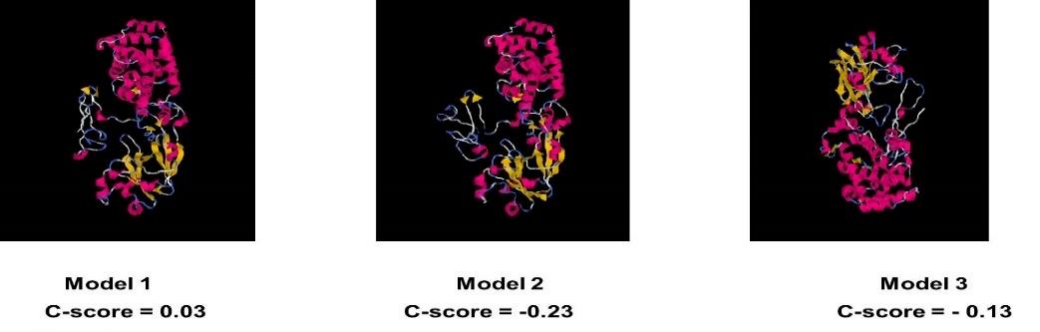
Tertiary structure prediction. A probabilistic structural model for DT390-STxB chimeric protein by ITASSER server. Based on C-scores, the model 1 has a high confidence score among other models

Secondary and tertiary structures of chimeric protein were predicted to define the final structure of a chimeric protein; the DT390-STxB chimeric protein was submitted to I-TASSER and GOR4 online web tools. According to C-scores planned through this software, model 1 with a C-score of 0.03 had the maximum confidence among the other models. This was graphically signified in Figure 3. 

**Figure 4:**
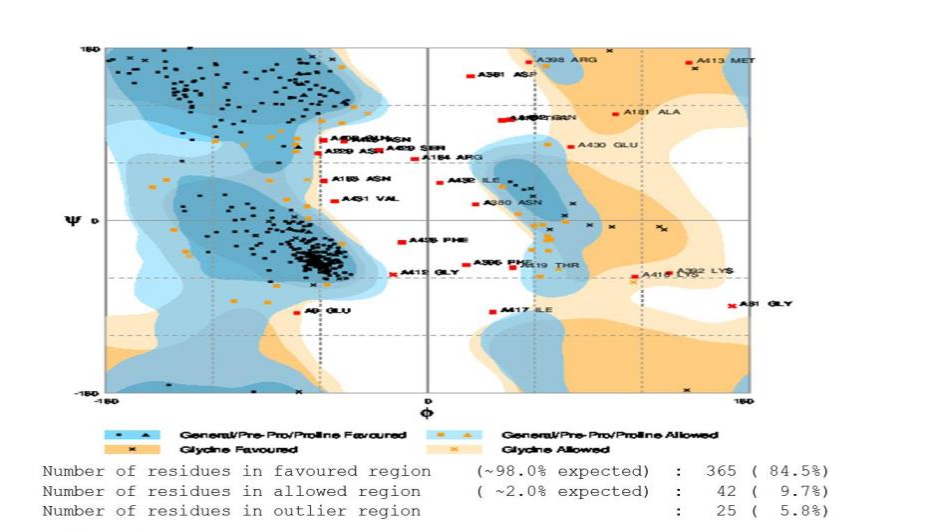
Evaluation of model stability is based on a Ramachandran plot for the DT390-STxB chimeric protein

The profile of energy minimization was considered by Swiss-PdbViewer –7107.159 kcal/mol, which demonstrated that the recombinant protein DT390-STxB had the suitable stability. Also, the structural stability of the protein was evaluated based on the data obtained from the Ramachandran plot (Figure 4).

## Discussion

Cancer is the most common cause of death in many developed countries. Today, the targeted therapies like the use of immunotoxins are increased which target specific antigens or receptors on the surface of tumor cells. Immunotoxins are recombinant proteins, which consist of a tumor-specific ligand-like antibody and a toxic protein. Ligand-targeted therapy makes it possible for tumor specificity and limited toxicity and promises for developing novel therapies for cancer treatment. It can carry higher doses of a drug to the tumor tissue and may overcome obstacles presented by cytotoxic chemotherapy (33-40).

Native DT is a 538-amino acid protein consisting of three domains. Part A is the enzymatically active domain, part B has a hydrophobic domain at the N-terminal portion, and the C-terminal portion of fragment B is the receptor-binding domain. In this study, we evaluated DT390-STxB chimeric protein as a new anti-tumor candidate via genetically fusing the DT390 fragment of DT538 to STxB (41-43).

In a study carried out by Imani-Fooladi *et al.*, the genetically fused protein called the TGFαL3-SEB fusion protein was designed and evaluated as a novel anti-tumor candidate. This protein was constructed by fusing the third loop of transforming growth factor alpha (TGFαL3) to the staphylococcal enterotoxin type B. The CAI index of the TGFαL3-SEB fusion protein enhanced from 0.5 in the wild type to 0.85 in the chimeric optimized gene. Moreover, the overall guanine-cytosine (GC) content decreased from 45.83% to 44.06%, hence increasing the overall stability of mRNA of the synthetic gene. The random coil was the second most available structure in this experiment. The pI of the hydrophilic TGFαL3-SEB fusion protein was 7.72. The highest C-score of -0.42 was annotated for tertiary structure of the TGFαL3-SEB protein (44). The comparison between the two chimeric proteins showed that the TGFαL3-SEB and DT390-STxB constructs are stable enough and have sufficient affinity to the overexpressed cancer cell receptors. 

In another study by Keshtvarz *et al.*, PE38-P4A8 chimeric immunotoxin was designed and evaluated. The GC content and codon bias of both wild and synthetic form of the protein were assessed and the results for optimized gene showed no rarely used codon, and the codon bias was demonstrated in *E. coli* as the bacterial host. The optimized GC content was about 54.2% and the CAI value was 0.94 revealing the high and stable expression in bacterial cells. The highest C-score of -3.36 observed in tertiary structure of the fusion protein. For isolating two parts of the proteins, the ASGGPE and (G4S)_3 _linkers were recruited (45). 

*In silico* studies are able to complete trans-criptional and translational gene fusion, other than the quality expression of the suggested concept in host expression vectors. CAI is the main factor used for gene optimization within a range of 0-1 and an ideal value of 1.0. Subsequently, our objective was to construct a fusion protein that is expressed in *E. coli* as a host expression vector. So, the codon table of *E. coli* was selected for back-translation of the amino acid sequence and ideal expression of the construct. In our gene, CAI index was improved from 0.6 in the wild type sequences to 0.9 in the chimeric optimized gene. Furthermore, the overall GC content was reduced from 50.03% to 47.58%, which should increase the overall stability of mRNA from the synthetic gene. Codon optimization contributes to improve the expression of the synthetic construct in the desired host vector.

The mRNA structure optimization was established based on the low ΔG value and the MFE values of the start codon. These characteristics can support ribosome binding and translation initiation. For prediction of RNA secondary structure, a genetic algorithm-based approach along with relative sequence analysis was recruited to define the prospective folding of the chimeric gene. The 5' terminus of the gene was folded in a way which is typical for all bacterial gene structures. The MFE for secondary structures formed by RNA molecules was also predicted. The mRNA secondary structure of the chimeric gene was analyzed using the mfold program with the parameters as follows: linear RNA folding at 5%, window=20, and max folds=50. All 32 structures achieved in this analysis exposed folding of the RNA construct at 37°C with initial ΔG ranging from -543.80 to -481.78 kcal/mol. The best structure (ΔG=-427.40 kJ/mol) is shown in Figure 4. The data showed that the mRNA was stable enough for proper translation in the new host.

ProtParam was used to determine the physio-chemical properties of the protein sequence. The results of the primary structure analysis showed that DT390-STxB fusion protein is hydrophilic in nature due to the large number of polar residues. Also, the primary structure analysis suggests that the average molecular weight of DT390-STxB is considered to be 53.64 kDa. The pI of a protein is defined as the pH at which the net charge on the surface of the protein is zero. At pI, the proteins are stable and dense. The calculated pI value of DT390-STxB is 5.11 (pI<7). Although ExPASy’s ProtParam computes the extinction coefficient (ɛ) for a range of wavelengths (276, 278, 279, 280, and 282 nm), extinction constant (K) of DT390-STxB at 280 nm is 49850 M^–1^ cm^–1^ with respect to the low concentration of Cys, Trp, and Tyr, which indicates that this fusion protein cannot be analyzed using UV spectral methods. 

The three-dimensional (3D) structure of the proteins is of a major importance to provide insights into their molecular functions. The 3D model of the recombinant DT390-STxB protein was produced using the I-TASSER online server, which generates 3D models along with their C-score, Z-score, RMSD, and TM-score. This server was generated in three models with C-scores as follows: -2.82, -3.32, and 0.03. Among the three models, model one was selected for further analysis as it assigned the highest C-score (0.03). The expected TM-score was 0.72±0.11, which approved the validity of the model. A TM-score more than 0.5 probabilities accuracy of topology. Subsequently, by generating 3D model, structural assessment and stereo chemical analyses were accomplished using Procheck Ramachandran plot. Energy minimization was determined by analysis of 3D structural stability of the chimeric protein using Swiss-PdbViewer. The proportion of residues was 84.5% in the favored region, 9.7% in allowed region, and 5.8% in the outlier region. Also, the assessment of model stability of the Ramachandran plot indicated that the most residues of the chimeric model are in a stable zone. The model was analyzed by different DT390-STxB fusion proteins as a new anti-tumor candidate. Since it is important to establish the structure-function relation of DT390-STxB fusion protein, some experimental studies have already started investigating the DT390-STxB fusion protein by several tools and software. 

In this study, we introduce a novel anti-tumor fussion protein. Our bioinformatics results show that this protein was a stable chimeric protein and it can be used for treatment against breast cancer.
